# *Ano5* Deficiency Leads to Abnormal Bone Formation via miR-34c-5p/KLF4/β-Catenin in Gnathodiaphyseal Dysplasia

**DOI:** 10.3390/ijms26115267

**Published:** 2025-05-30

**Authors:** Shengnan Wang, Shuai Zhang, Huichong Xu, Mingyue Zhang, Xiu Liu, Sirui Liu, Hongyu Li, Ying Hu

**Affiliations:** Beijing Institute of Dental Research, Beijing Stomatological Hospital, Capital Medical University, No.9 Fanjiacun Road, Fengtai District, Beijing 100070, China; wangsn1101@yeah.net (S.W.); zskqdoctor@126.com (S.Z.); xhc000831@163.com (H.X.); 15046468812@163.com (M.Z.); liuxiu1206@foxmail.com (X.L.); liusirui1122@mail.ccmu.edu.cn (S.L.)

**Keywords:** GDD, *Ano5*, miR-34c-5p, osteoblast, KLF4

## Abstract

Gnathodiaphyseal dysplasia (GDD) is a rare autosomal dominant genetic disease, mainly characterized by enlargement of the mandible, osteosclerosis, and frequent fracture of tubular bone. GDD is caused by heterozygous mutations in *Anoctamin 5* (*ANO5*). We have previously generated an *Ano5* knockout (KO) mice model and validated the phenotypes consistent with GDD patients, including enhanced bone formation and alkaline phosphatase (ALP) activity. Experiments have identified that *Ano5* deficiency elevated the osteogenesis of calvaria-derived osteoblasts (mCOBs). In this study, we found that *Ano5* deficiency notably inhibited miR-34c-5p expression. Krüppel-Like Factor 4 (*Klf4*), a target gene of miR-34c-5p confirmed by dual luciferase reporter assay, was up-regulated in *Ano5^−/−^* mCOBs, accompanied by activated downstream canonical Wnt/β-catenin signaling and increased expression of β-catenin. Overexpression of miR-34c-5p in *Ano5^−/−^* mCOBs inhibited osteogenic capacity by suppressing proliferative capacity, osteoblast-related factor levels, ALP activity, and matrix calcification through regulating KLF4/β-catenin signaling axis. Furthermore, miR-34c-5p adeno-associated virus (AAV) treatment in vivo rescued the abnormally thickened cortical bone and enhanced biomechanical properties in *Ano5*^−/−^ mice. Importantly, the serum level of P1NP, a marker of bone formation, was also significantly declined. We conclude that dysregulation of miR-34c-5p contributes to the enhanced osteogenesis in GDD by excessive activation of KLF4/β-catenin signaling axis under Ano5-deficient conditions. This study elucidates the pathogenesis of GDD and provides novel insights into the therapeutic strategies.

## 1. Introduction

The Gnathodiaphyseal dysplasia (GDD; OMIM: 166260) is a systemic disorder of bone metabolism, characterized by abnormal enlargement and ossifying fibroma of the mandible. Patients experienced increased thickening of the long bone cortex and frequent fractures [1,2]. Using gene linkage analysis, Tsutsumi determined that the *Anoctamin5 (ANO5)* was the pathogenic gene of GDD [3]. ANO5, also referred to as TMEM16, is a 913-amino acid transmembrane protein located in the endoplasmic reticulum and cytomembrane, functioning as a Ca^2+^-dependent chloride channel and phospholipid scramblase [4]. It is intimately linked to inflammation, lipid metabolism, and sperm motility.

In order to study the mechanism of the GDD, our group generated a knock-in model to study the heterozygous mutation in *ANO5* in a Chinese GDD family (c.1079G>A; p. Cys360Tyr) [1,5]. The metabolomics and transcriptomics analyses showed *Ano5^Cys360Tyr^* mutation disturbs calcium homeostasis and cellular proliferation of osteoblasts [6]. Different *ANO5* mutations associated with GDD have been reported [7,8]. Therefore, based on the hotspot region of mutation, our group also generated an *Ano5* knockout mice model by deleting 833 bp targeting exons 11 and 12. Similar to patients, the *Ano5*^−/−^ mice displayed enlarged mandibles, enhanced bone density, thickened bone cortex, and elevated serum ALP level. The osteogenic ability of the *Ano5*^−/−^ mCOBs was also upregulated, which further implies that enhanced bone formation is a key pathological feature of GDD [9].

There is growing evidence that microRNAs (miRNAs) are linked to many bone disorders and regulate different phases of bone development [10]. miRNAs are a class of small non-coding RNAs, approximately 20–24 nucleotides in length [11]. They participate in the post-transcriptional regulation of gene expression in animals, plants, and various viruses [12]. The 5′ end of the mature miRNA binds to the 3′ end of the target mRNA through base pairing, leading to mRNA instability and translational repression [13]. miR-34c-5p is a member of the miR-34 family and has been implicated in the regulation of cardiac hypertrophy, lung cancer metastasis, and osteogenic process [14,15,16]. According to some studies, miR-34c-5p targets LGR4 to activate the function of osteoclasts, which affects bone formation [17]. It also regulates Notch signaling, which results in decreased osteoblast differentiation and increased bone resorption via elevated osteogenesis [18].

Krüppel-Like Factor 4 (KLF4) is an evolutionarily conserved transcription factor, containing a zinc finger structure, and is widely expressed in monocytic cells and various tissues, including skin, intestine, testis, lung, teeth, and bone [19]. It is involved in numerous biological processes, including differentiation, proliferation, development, and tissue homeostasis [20]. The carboxyl-terminal of KLF4 has a DNA-binding structural region that contains three Cys2His2 (C2H2) type zinc finger structures, which are formed by 81 highly conserved amino acids. It regulates transcription through binding to CACCC and GC-rich elements in target gene DNA sequences [21]. An activation domain and a repression domain exist in the amino terminus of KLF4 [22]. Currently, the biological roles of KLF4 have been studied in breast cancer, colon cancer, and other diseases [23]. Overexpression of KLF4 in osteosarcoma cells significantly enhanced the proliferation by activating the Wnt/β-catenin signaling pathway [24]. KLF4 contributed the odontogenic cells differentiation through activating the TGF-β signal pathway [25]. The proliferation and osteogenic differentiation of BMSCs were promoted by the activation of KLF4/SOX2/IGF2 axis [26]. However, some studies showed that KLF4 repressed osteoblast differentiation via Hedgehog signaling [27]. The roles and mechanisms of KLF4 on bone homeostasis require further investigation, and the relationship between KLF4 and GDD remains to be verified.

In the current study, *Ano5* deficiency reduced the expression of miR-34c-5p in mCOBs. We found that miR-34c-5p directly targeted the 3′UTR region of *Klf4* and affected β-catenin signaling, which clarified the important mechanism of *Ano5* deficiency leading to enhanced osteogenic ability of mCOBs. Partial abnormal bone phenotypes of GDD were rescued by local injection of AAV9-miR-34c-5p into the mandible and long bone. This study proposed new ideas for the clinical management of GDD.

## 2. Results

### 2.1. miR-34c-5p Was Down-Regulated in the Ano5^−/−^ mCOBs

**Figure 1:**
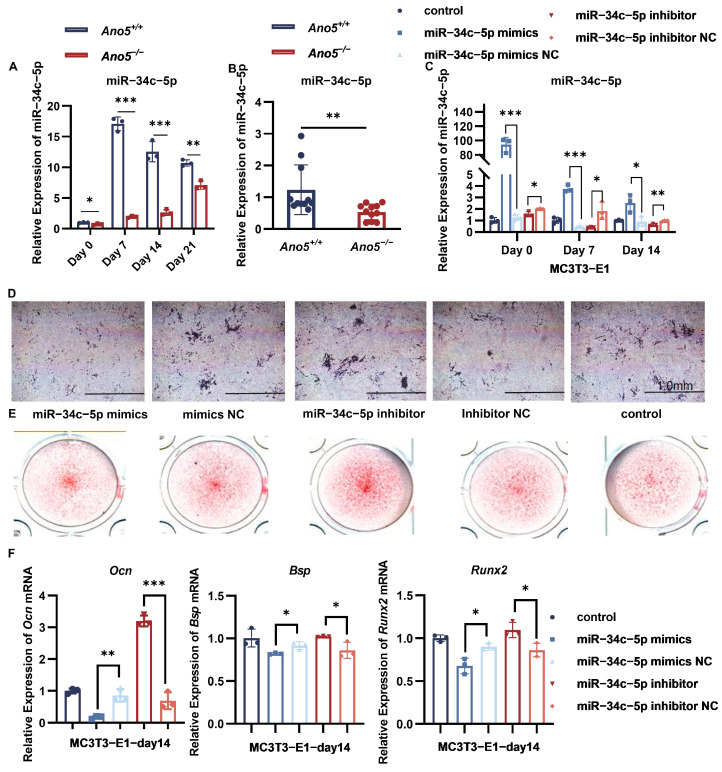
miR-34c-5p was down-regulated in the *Ano5^−/−^* mCOBs. (**A**) The expression of miR-34c-5p in mCOBs was examined using qRT-PCR. (**B**) The expression of miR-34c-5p in 16-week-old male mouse femurs was examined using qRT-PCR. (**C**) MC3T3-E1 was transfected with miR-34c-5p mimics or inhibitor followed by osteogenic introduction. (**D**,**E**) ALP staining was examined 14 days after osteogenic treatment and Alizarin red staining was examined after 21days. (**F**) The mRNA levels of osteogenic factors were analyzed using qRT-PCR. * *p* < 0.05, ** *p* < 0.01 and *** *p* < 0.001.

Our previous research has demonstrated that enhanced osteogenesis is a vital phenotype of GDD, but the mechanism remains unclear. miR-34c-5p is tightly associated with osteogenic differentiation and is expressed in human periodontal ligament stem cells, osteoclasts, and bone marrow mesenchymal stem cells (BMSCs) [28]. Here, we examined the expression of miR-34c-5p in osteoblasts and femurs of 16-week-old mice. During osteogenesis, *Ano5*^−/−^ mCOBs showed a considerably lower expression of miR-34c-5p than *Ano5*^+/+^ mCOBs (Figure 1A). In femurs, *Ano5* deficiency similarly resulted in decreased expression of miR-34c-5p (Figure 1B).

We investigated the connection between miR-34c-5p and osteogenic differentiation in MC3T3-E1. Consequently, lentivirus progressively modified the expression of miR-34c-5p in MC3T3-E1 (Figure 1C). Briefly, compared with the negative control (NC), overexpression of miR-34c-5p decreased the alkaline phosphatase (ALP) activity and the formation of calcium nodules in MC3T3-E1 (Figure 1D,E). It down-regulated the mRNA levels of osteogenesis-related genes, including osteocalcin (*Ocn*), runt-related transcription factor 2 (*Runx2*), and bone sialoprotein (*Bsp*) (Figure 1F). While the suppression of miR-34c-5p in MC3T3-E1 promoted osteogenic differentiation. These results further confirmed the negative regulation of miR-34c-5p on osteogenesis.

### 2.2. miR-34c-5p Rescued the Abnormal Osteogenic Ability of Ano5^−/−^ mCOBs

**Figure 2:**
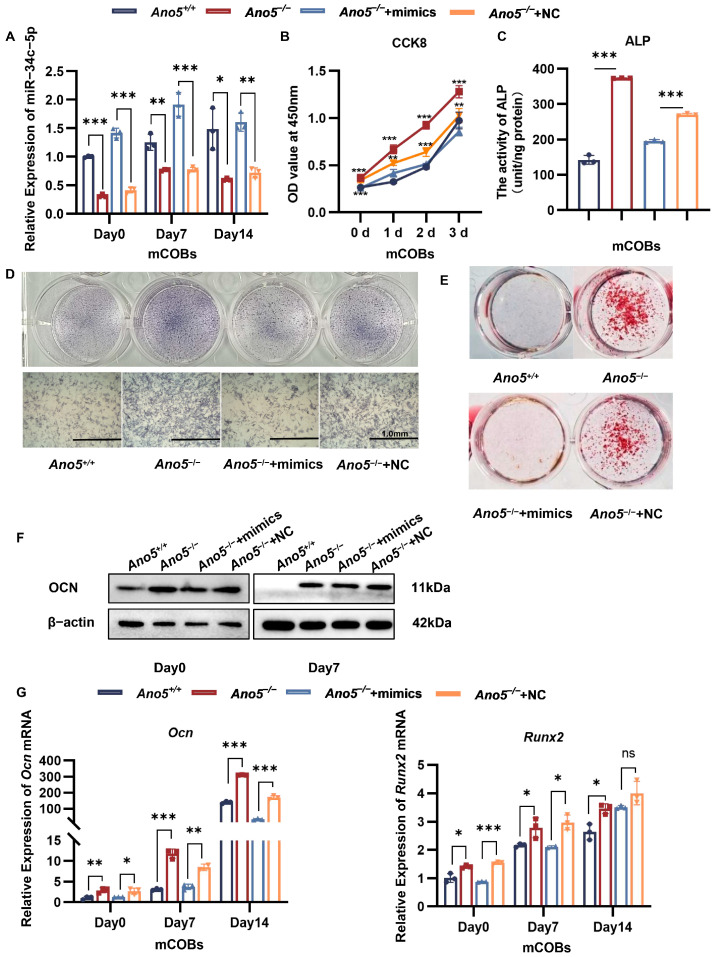
miR-34c-5p rescued the abnormal osteogenic ability of *Ano5^−/−^* mCOBs. (**A**) mCOBs were transfected with miR-34c-5p mimics or negative control (NC) followed by osteogenic introduction. (**B**) Cellular proliferation was evaluated using CCK-8 assays. (**C**,**D**) ALP staining and activity were examined 5 days after osteogenic induction. (**E**) Alizarin red staining was examined 21 days after osteogenic treatment. (**F**) The protein levels of OCN were analyzed using Western blot. (**G**) The mRNA levels of *Ocn* and *Runx2* were analyzed using qRT-PCR. * *p* < 0.05, ** *p* < 0.01, *** *p* < 0.001 and ns: no significance.

We used lentivirus to increase the expression of miR-34c-5p in *Ano5*^−/−^ mCOBs to detect the relationship between miR-34c-5p and enhanced bone formation in GDD (Figure 2A). The proliferative capacity in *Ano5*^−/−^ mCOBs was abnormally built up, while it was inhibited by miR-34c-5p (Figure 2B). Additionally, overexpression of miR-34c-5p in *Ano5*^−/−^ mCOBs decreased the ALP activity and the number of mineralized nodules (Figure 2C–E). The miR-34c-5p treatment reversed the *Ocn* and *Runx2* mRNA levels (Figure 2G), and decreased the protein level of OCN (Figure 2F, Supplemental Figure S4A). All things considered, overexpression of miR-34c-5p in *Ano5^−/−^* osteoblasts rescued the excessive osteogenic differentiation, which implied the declined expression of miR-34c-5p is responsible for the abnormal bone formation of GDD.

### 2.3. miR-34c-5p Directly Targeted KLF4

**Figure 3:**
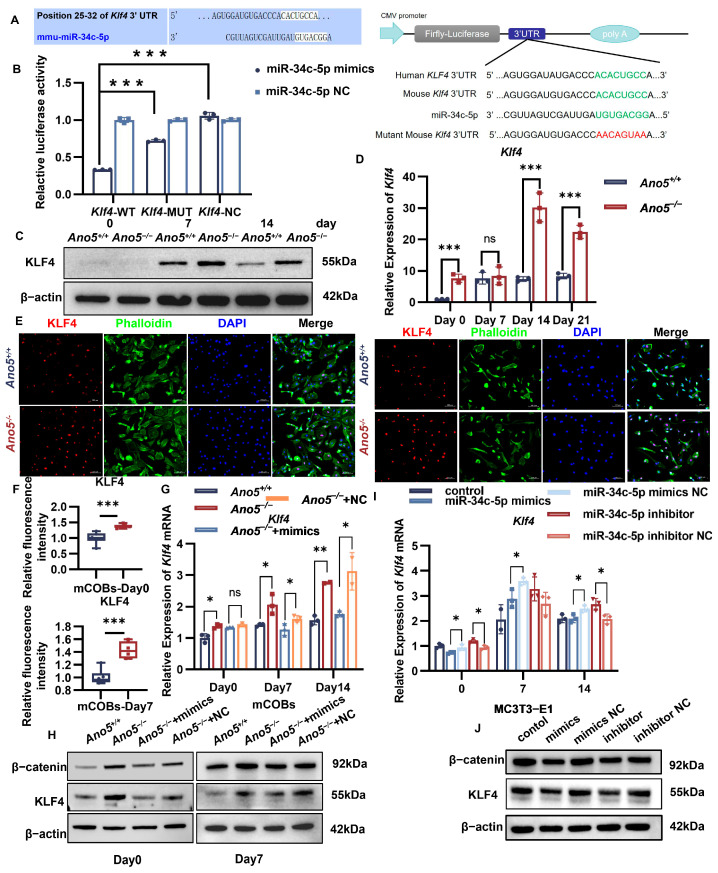
miR-34c-5p directly targeted to KLF4. (**A**) TargetScan predicted that the 3’UTR of *Klf4* was targeted by miR-34c-5p. (**B**) The relative luciferase activity of wildtype or mutant construct of *Klf4* 3′-UTR was analyzed in HEK-293t transfected with negative control (NC) or miR-34c-5p mimics. The expression of KLF4 in mCOBs during osteogenic induction was analyzed using Western blot (**C**), qRT-PCR (**D**), and Immunofluorescence (**E**,**F**). (**G**) The mRNA levels of *Klf4* were examined when treated with miR-34c-5p mimics. (**H**) The expression of KLF4, β-catenin in mCOBs was examined when treated with miR-34c-5p mimics. (**I**) The mRNA levels of *Klf4* were examined when treated with miR-34c-5p mimics or inhibitor. (**J**) The expression of KLF4, β-catenin in MC3T3-E1 was examined when treated with miR-34c-5p mimics or inhibitor. * *p* < 0.05, ** *p* < 0.01, *** *p* < 0.001, and ns: no significance.

According to the TargetScan database, *Klf4* is one of the most significant targeted genes of miR-34c-5p (Figure 3A). A dual-luciferase activity test was used to further test if miR-34c-5p directly targets the 3′-UTR of *Klf4*. The findings demonstrated that miR-34c-5p overexpression reduced the luciferase activity of *Klf4* 3′-UTR construct in HEK-293t cells compared with the negative control group. When the putative binding mutant sequence was transfected, this inhibitory effect was partly eliminated (Figure 3B). These results suggest that miR-34c-5p inhibits the transcription of *Klf4* by directly targeting its 3′-UTR region.

As determined by qRT-PCR and Western blot, KLF4 expression was higher in the *Ano5^−/−^* osteoblasts than in the *Ano5^+/+^* group (Figure 3C,D, Supplemental Figure S4B). KLF4 is a transcription factor that is crucial for controlling cell division and proliferation. Immunofluorescence results showed that KLF4 was located in the nuclei of mCOBs either non-osteogenic or osteogenic induction, and the fluorescence intensity in *Ano5^−/−^* osteoblasts was increased (Figure 3E,F). We then observed the regulatory effect of miR-34c-5p on KLF4. Importantly, qRT-PCR and Western blot demonstrated that miR-34c-5p overexpression could decline KLF4 expression in *Ano5*^−/−^ mCOBs compared with the negative control (NC) group (Figure 3G,H, Supplemental Figure S4C). The decline of the expression of KLF4 appeared in MC3T3-E1 with miR-34c-5p overexpression. The down-regulation of miR-34c-5p in MC3T3-E1 up-regulated the *Klf4* mRNA level compared with the NC group, though the changes were not enough to affect the protein level of KLF4 (Figure 3I,J, Supplemental Figure S4D).

### 2.4. Inhibiting KLF4 Attenuated Abnormally Enhanced Osteogenesis of Ano5^−/−^ mCOBs

**Figure 4:**
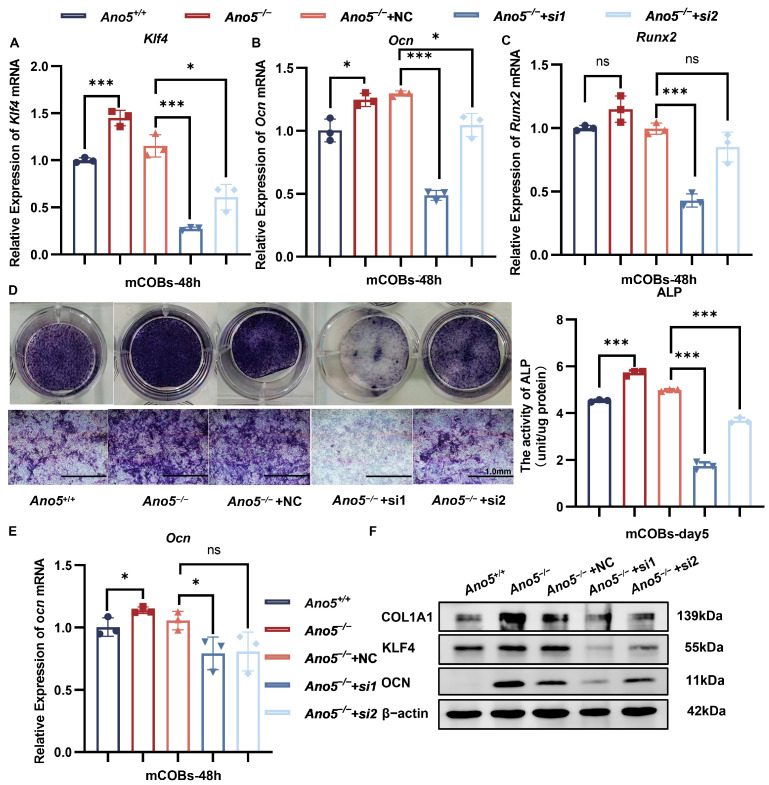
Inhibiting KLF4 reduced abnormally enhanced osteogenesis of *Ano5^−/−^* mCOBs. (**A**–**C**) The mRNA levels of *Klf4*, *Ocn*, *Runx2* in mCOBs were analyzed 48 h after siKLF4 transfection using qRT-PCR. (**D**) ALP staining and activity were examined 5 days after osteogenic treatment. (**E**,**F**) The mRNA and protein levels of osteogenic factors were analyzed using qRT-PCR and Western blot. * *p* < 0.05, *** *p* < 0.001, and ns: no significance.

We used siRNA to reduce KLF4 expression in *Ano5*^−/−^ osteoblasts in order to examine the impact of KLF4 dysregulation on the increased osteogenesis in GDD. qRT-PCR showed the expressions of *Klf4* were reduced by more than 50% at 48 h after transfection (Figure 4A). In the meantime, the expression of *Ocn* and *Runx2* was declined (Figure 4B,C). During osteogenesis induction, the knockdown of KLF4 was sufficient to decrease the ALP activity of *Ano5*^−/−^ mCOBs to corresponding levels in the wild-type group (Figure 4D). Additionally, the expressions of OCN and COL1α1 were rescued to the normal levels following KLF4 knockdown in *Ano5*^−/−^ osteoblasts (Figure 4E,F, Supplemental Figure S4E). Considering the therapeutic effect of inhibiting KLF4 on the osteogenesis of *Ano5*^−/−^ mCOBs, we speculated that the miR-34c-5p/KLF4 signaling pathway plays a vital role in the abnormal bone metabolism in GDD.

### 2.5. Ano5 Deficiency Upregulated β-Catenin Signaling Pathway

**Figure 5:**
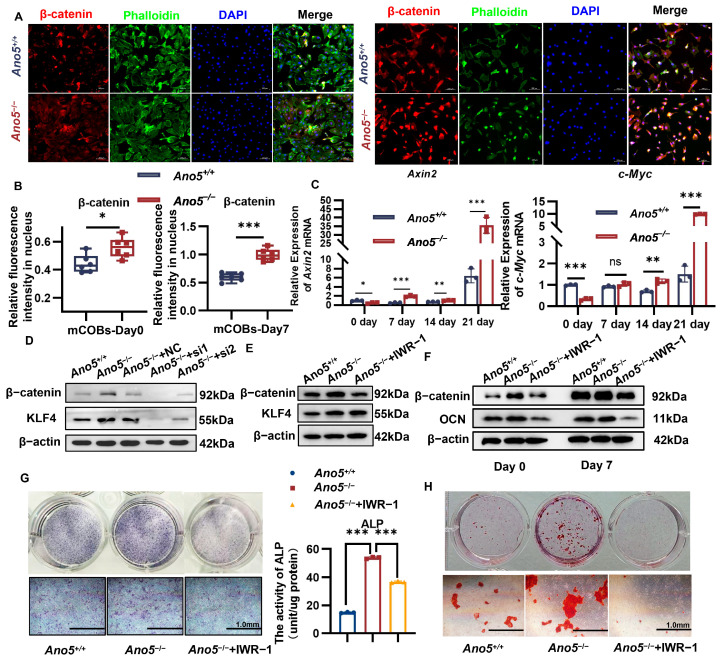
miR-34c-5p/KLF4 regulated the β-catenin pathway and influenced osteogenic differentiation. (**A**) The Immunofluorescence showed the expression and location of β-catenin in mCOBs. (**B**) The positive expression of β-catenin in the nucleus of mCOBs. (**C**) The expression of β-catenin pathway relevant downstream factors was analyzed using qRT-PCR. (**D**) The expression of β-catenin in mCOBs with siKLF4 treatment was analyzed using Western blot. (**E**) The expressions of KLF4 in mCOBs with IWR-1 treatment were analyzed using Western blot. (**F**) The expression of OCN in mCOBs with IWR-1 treatment after osteogenic induction was analyzed using Western blot. (**G**) ALP staining and activity were examined 5 days after osteogenic treatment. (**H**) Alizarin red staining was examined 21 days after osteogenic treatment. * *p* < 0.05, ** *p* < 0.01, *** *p* < 0.001 and ns: no significance.

KLF4 is engaged in several biological processes, including the Notch, Hedgehog, and Wnt/β-catenin pathways. Notably, the β-catenin signaling pathway is closely associated with bone formation. Immunofluorescence showed that *Ano5* deficiency increased β-catenin expression (Figure 5A,B, Supplemental Figure S1A). Notably, after osteogenic induction, more β-catenin located in the nucleus, and the fluorescence intensity in *Ano5*^−/−^ mCOBs was higher than the *Ano5*^+/+^ group. At different phases of osteogenic induction, the expressions of downstream genes of β-catenin signaling in the *Ano5*^−/−^ group, such as *Axin2* and *c-Myc*, were increased (Figure 5C). *Axin2* plays an important role in the regulation of the stability of β-catenin. *c-Myc* encodes a nuclear phosphoprotein regulating cell cycle progression, apoptosis, and cell differentiation [29]. These results indicate that *Ano5* deficiency resulted in the abnormal activation of the β-catenin signaling pathway in mCOBs.

### 2.6. miR-34c-5p/KLF4 Regulated the β-Catenin Pathway and Influenced Osteogenic Differentiation

Subsequently, we observed the regulatory effect of miR-34c-5p/KLF4 on the β-catenin signaling pathway. Overexpression of miR-34c-5p in MC3T3-E1 reduced the expression of β-catenin (Figure 3J, Supplemental Figure S4D), implying that β-catenin signaling is regulated by miR-34c-5p. miR-34c-5p also restored the abnormal elevation of β-catenin in *Ano5*^−/−^ mCOBs, though the difference was not obvious on the seventh day (Figure 3H, Supplemental Figure S4C). At the same time, we observed that KLF4 knockdown significantly decreased β-catenin expression in *Ano5^−/−^* mCOBs after 5 days of osteogenic induction (Figure 5D, Supplemental Figure S4F).

To demonstrate the impact of β-catenin excessive activation regulated by miR-34c-5p/KLF4 signaling on enhanced bone formation in GDD, *Ano5*^−/−^ mCOBs were exposed to IWR-1. IWR-1 inhibits β-catenin from entering the nucleus by inducing the AXIN2 up-regulation and stabilizing the β-catenin destruction complex [30]. In combination with the CCK8 assay, the 3 µM concentration was selected for subsequent experiments (Supplemental Figure S1B,C). Western blot results verified the sustained inhibitory effect of IWR-1 on β-catenin after seven days of osteogenic induction, but there was little effect on KLF4 expression (Figure 5E, Supplemental Figure S4G). IWR-1 also reduced the protein expression of OCN in *Ano5^−/−^* mCOBs at various phases of osteogenic induction (Figure 5F, Supplemental Figures S1D and S4H). Simultaneously, IWR-1 restored the excessive ALP activity (Figure 5G) and mineralization capacity of *Ano5^−/−^* mCOBs (Figure 5H). These results confirm that the abnormally enhanced osteogenic capacity of *Ano5^−/−^* mCOBs can be successfully restored by blocking the β-catenin signaling pathway.

### 2.7. AAV9-miR-34c-5p Rescued Part of the Bone Phenotype in Ano5^−/−^ Mice

**Figure 6:**
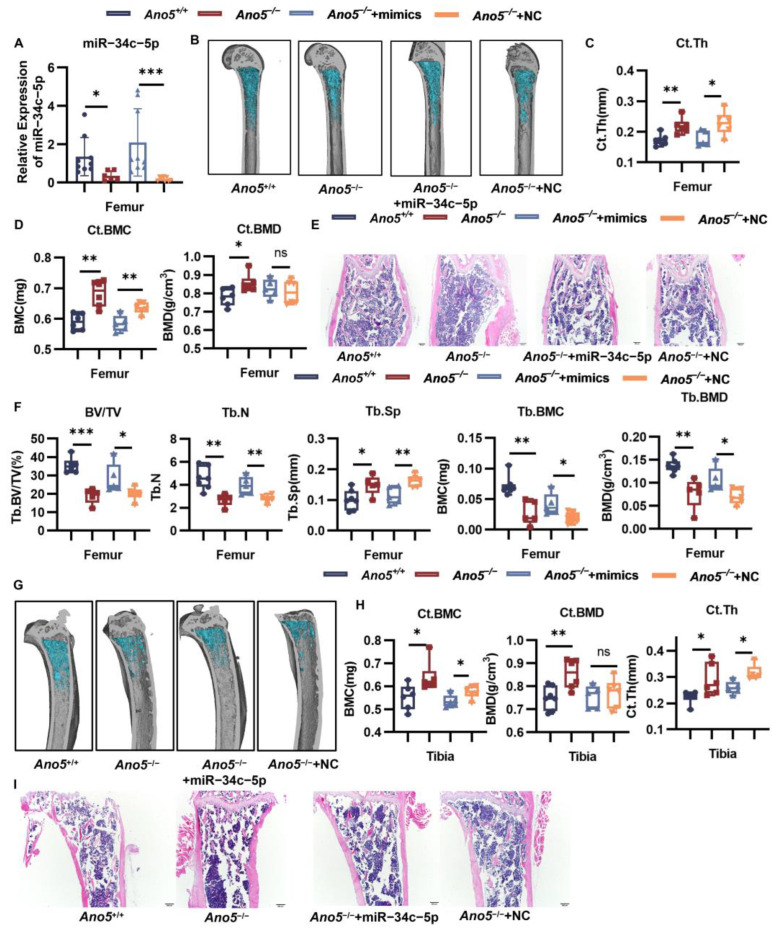
AAV-miR-34c-5p injection partially rescued abnormal bone formation of GDD in vivo. (**A**) The transfection efficiency of AAV-miR-34c-5p in femoral tissue was assessed by qRT-PCR. (**B**) Three-dimensional reconstructions of femurs were performed using Mimics Medical 21.0. (**C**,**D**) The femur-relative parameters were analyzed, including the BMC, BMD, and thickness of the cortex. (**E**) The paraffin sections of the mouse femur were subjected to H&E staining (The sections were imaged under light microscope with×40 magnification). (**F**) The BMC, BMD, number, and separation of the trabecula were analyzed. (**G**) Three-dimensional reconstructions of tibias were performed using Mimics Medical 21.0. (**H**)The tibia-relative parameters were analyzed, including the BMC, BMD, and thickness of the cortex. (**I**) The paraffin sections of the mouse tibia were subjected to H&E staining (The sections were imaged under light microscope with × 40 magnification). Data were presented as box plots with an indication of the median; whiskers represented min to max values and showed all points. Each dot represented a single animal. *n* = 7 for *Ano5*^+/+^ mice, *n* = 6 for *Ano5*^−/−^ mice, *n* = 5 for *Ano5*^−/−^ + miR-34c-5p mice, *n* = 6 for *Ano5*^−/−^ + negative control mice. * *p* < 0.05, ** *p* < 0.01,*** *p* < 0.001 and ns: no significance.

**Figure 7:**
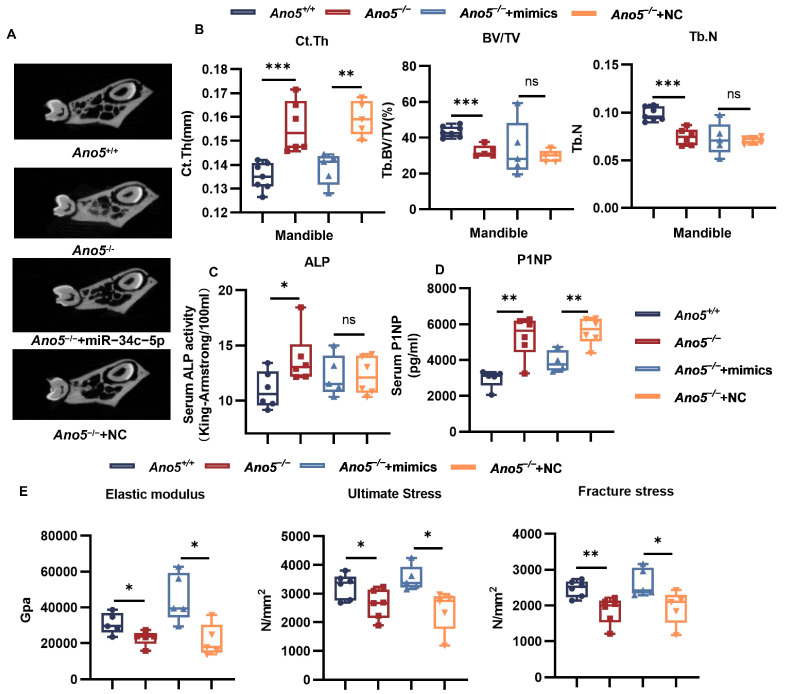
AAV-miR-34c-5p inhibited mandible bone formation and improved the mechanical properties in mice. (**A**) micro-CT images showed the mandible at the first molar. (**B**) The mandible-related parameters were analyzed, including the thickness, the trabecula numbers and the BV/TV of the mandible. (**C**,**D**) Serum ALP and P1NP were measured. (**E**) Three-point bending test and biomechanical property were analyzed. Data were presented as box plots with an indication of the median; whiskers represented min to max values and showed all points. Each dot represented a single animal. *n* = 7 for *Ano5*^+/+^ mice, *n* = 6 for *Ano5*^−/−^ mice, *n* = 5 for *Ano5*^−/−^ + miR-34c-5p mice, *n* = 6 for *Ano5*^−/−^ + negative control mice. * *p* < 0.05, ** *p* < 0.01, *** *p* < 0.001, and ns: no significance.

Adeno-associated virus (AAV) has distinct virtues in vivo, including high targeting, high titer, single injection required, and low immunogenicity. We observed the influence of AAV9-miR-34c-5p on abnormal bone phenotypes of GDD. After eight weeks of treatment, qRT-PCR showed that the AAV-miR-34c-5p virus increased the expression of miR-34c-5p in the femoral tissues of mice by eight times compared with the NC group (Figure 6A). The infection efficiency was also confirmed by obvious green fluorescence in the AAV-miR-34c-5p group (Supplemental Figure S2A,B). Previous research has reported the thickened cortical bone and decreased trabecular bone in *Ano5*^−/−^ mice. Of note, compared with the negative control, AAV9-miR-34c-5p decreased the thickness and bone mineral content (BMC) of the femurs’ cortical bone but did not change the bone mineral density (BMD) (Figure 6B–D). H&E staining and quantitative analysis of the femur also showed that miR-34c-5p reduced the thickness of cortical bone of *Ano5*^−/−^ mice to the level of *Ano5*^+/+^ mice (Figure 6E, Supplemental Figure S2C). miR-34c-5p rescued the bone volume fraction (BV/TV) and BMD of trabecular, trabecular numbers and separation to comparable levels to *Ano5*^+/+^ mice (Figure 6F). The treatment resulted in a decreased tendency to the BMC and thickness of the tibias’ cortical bone (Figure 6G–I, Supplemental Figure S2D), though there was no significant difference for the BMD of the tibia cortex and trabecular (Supplemental Figure S2E–I). In the mandible, the thickness of the lingual cortex was decreased, though the trabecular failed to respond to AAV9-miR-34c-5p (Figure 7A,B).

Alkaline phosphatase level in the serum reflected the metabolic capacity of bone. *Ano5* deficiency also increased the ALP content in serum, but the AAV9-miR-34c-5p treatment failed to rescue the high ALP level (Figure 7C). P1NP is the N-terminal peptide of type I collagen produced by osteoblasts. With the synthesis of new type I collagen in bone formation, protease will cut P1NP from type I procollagen and release them into the bone matrix and blood circulation. Therefore, serum P1NP level is a classical marker of new bone formation [31]. *Ano5* deficiency increased the P1NP content in the mice, which was attenuated by the AAV9-miR-34c-5p injection (Figure 7D).

Patients with GDD are prone to fracture of long bones, and *Ano5*^−/−^ mice showed increased bone fragility. The three-point bending force test showed that the treatment of AAV9-miR-34c-5p for *Ano5*^−/−^ mice improved the fracture stress and ultimate stress, and the elastic modulus of the tibia was increased markedly (Figure 7E), though didn’t change the fracture force and ultimate force of the tibia (Supplemental Figure S3A,B).

Therefore, our study reveals that *Ano5* knockout led to significant down-regulation of miR-34c-5p, which excessively activates the β-catenin signaling pathway by directly targeting KLF4. Overexpression of miR-34c-5p can rescue abnormal enhanced bone formation of GDD in vitro and in vivo.

## 3. Discussion

GDD is characterized by skeletal abnormalities that mostly affect the long bones and jaw, with abnormal cortical bone thickening and increased serum ALP levels [2,3,4]. *ANO5* gene mutations at various locations have been extensively documented in GDD patients [1]. We have successfully created an *Ano5*^−/−^ mice model to investigate the etiology of GDD, which aligns with the clinical phenotypes in GDD patients [7]. In this study, we found that *Ano5* knockout suppressed the expression of miR-34c-5p in mouse bone tissue and osteoblasts, which in turn promoted the expression of the target gene *Klf4*. Then the up-regulation of KLF4 activated β-catenin signal and led to an abnormal enhancement of the osteogenic capacity of *Ano5*^−/−^ osteoblasts.

Numerous studies show that miRNAs impact bone metabolisms [32,33]. miR-34c-5p inhibits osteogenic differentiation by targeting the Bcl-2 gene [34]. Prostaglandin E2-induced exosomes vesicles containing miR-34c-5p inhibited osteogenic differentiation of human periodontal ligament stem cells via the SATB2/ERK axis [28]. Importantly, we discovered that *Ano5* deficiency decreased the expression of miR-34c-5p in both osteoblasts and mouse femurs. Our group also found reduction of miR-34c-5p caused by *Ano5^Cys360Tyr^* mutation reported in a Chinses GDD family [1]. It is reported that overload of Ca^2+^ could activate the transcription factor Ap-1/c-Jun, which directly bind to miR-34c-5p to promote the transcription [35]. Additionally, PI3K-AKT could repress osteogenic differentiation through upregulating the expression of miR-34c-5p [36], while *Ano5* deficiency decreased the concentration of Ca^2+^ and inhibited AKT activation osteoblasts, further indicating a vital role of miR-34c-5p on GDD [37,38].

For the first time, we connected the pathophysiology of GDD to miR-34c-5p. Our study revealed that overexpression of miR-34c-5p in the *Ano5*^−/−^ mCOBs rescued the abnormal osteogenesis, including the ALP activity, mineralized ability, and *Ocn* level. After the AAV9-miR-34c-5p treatment, the excessively mineralized and thickened cortical bones were rescued partially, providing a sufficient development environment for the growth of trabeculae bones. Although there were no significant changes in the trabeculae bone of tibia, the strengthened biomechanical property of it implied the structure and morphology of the bone was improved. There are studies showing that abnormally thickened cortical bone did not improve bone hardness due to damage to the lacuno-canalicular network [39]. We speculated that miR-34c-5p restored the normal interior structure of the cortical bone, which contributes to normal bone developmental environment [40]. The temporomandibular joint of mice is difficult to inject, so the lingual cortex of the mandible of mice was chosen. Subsequently, the thickened lingual cortexes in *Ano5*^−/−^ mice were rescued. However, the effects of AAV on the lateral cortex and bone trabeculae were not significant, which may be related to the injection sites and the complex development process of the mandible, such as masticatory stimulus and anatomical morphology [41].

Overexpressing of miR-34c-5p restored ALP activity in the *Ano5*^−/−^ osteoblasts. However, there was no significant reduction of serum ALP level with AAV9-miR-34c-5p injection. ALP levels in serum are regulated by multiple organs, such as the liver, kidney, and gut, meaning that they may not be affected by the local injection of AAV into bone tissues. P1NP is a more specific and sensitive indicator of new bone formation as it is not affected by hormones [31]. miR-34c-5p decreased the P1NP content in serum, suggesting the abnormal osteogenesis in *Ano5*^−/−^ mice was restored.

In addition, our study firstly confirmed that miR-34c-5p could directly target 5′-ACACUGCC-3′ in the 3′UTR of *Klf4* to inhibit its transcription. We found that miR-34c-5p overexpression reduced the mRNA and protein levels of KLF4 both in MC3T3-E1 and mCOBs. The mutant *Klf4* that we established can only partially rescue the reduced luciferase activity caused by miR-34c-5p binding, suggesting other sites needed to be further explored. Researchers identified KLF4 as a BMP2-dependent transcription factor that acted synergistically with *Runx2* to achieve a regulatory effect on osteogenesis [42]. Furthermore, our results showed that *Ano5* deficiency increased KLF4 expression, while siKLF4 could attenuate excessively elevated osteogenic capacity in GDD. Conversely, KLF4 inhibits bone formation in mature osteoblasts from Col1α1-Cre mice [43], implying that KLF4 plays different roles in the early and mature osteoblasts, which was related to the bidirectional regulation of KLF4 [22]. It is reported that the knockdown of KLF4 in the Sp7+ lineage interferes with mandible development [44]. Notably, either *Ano5* knockout mice or GDD patients are manifested by enlargement of the mandible. Besides, our group previously found that elevated bone mass of GDD is attributed to reduced osteoclastogenesis which is partly affected by OPG/RANKL up-regulation secreted by osteoblasts [9]. RANKL derived from osteoblasts and osteocytes promotes osteoclast differentiation and maturation by binding to RANK receptors on the surface of osteoclasts and their precursors [45]. Studies have also shown that overexpression of KLF4 inhibits Rankl expression in osteoblasts, leading to impaired osteoclast differentiation and maturation, which is consistent with our reduced osteoclast function in GDD [43]. Our results implied that the up-regulation of KLF4 mediated by declined miR-34c-5p in osteoblasts was intimately linked to GDD.

KLF4 promotes the differentiation of smooth muscle cells during angiogenesis by activating Wnt/β-catenin [46]. Activating the Wnt/β-catenin signaling pathway leads to the translocation of β-catenin to the nucleus as a co-activator of TCF/LEF family member to regulate downstream genes [47]. Activation of β-catenin signaling improves osteogenesis and hematopoietic recovery after chemotherapy [48]. Another *Ano5*^−/−^ mice model (C57BL/6-*Ano5*<tm1Itak>) to GDD, which is different from our knockout site, showed low bone volume and defective osteoblast differentiation. In this study, researchers concluded that *Ano5* deficiency weakened Ca^2+^ homeostasis in bone metabolism, decreasing WNT/β-catenin signaling in osteoblasts [37]. In our study, *Ano5* deficiency aberrantly activated the β-catenin signaling pathway, and the β-catenin inhibitor rescued the abnormal osteogenesis, meaning that the activating β-catenin pathway played a role in the osteogenesis in GDD. Notably, when KLF4 was knocked down in *Ano5*^−/−^ osteoblasts, the β-catenin pathway was inhibited, suggesting that KLF4 may act on molecules upstream of β-catenin. However, KLF4 can induce Dickkopf-1 (DKK1), a negative regulator of the Wnt/β-catenin signaling pathway, to up-regulate transcription, which was contradicted by our results [49]. The hTFtarget website predicted KLF4 may target Glycogen synthase kinase-3β (GSK-3β), a negative regulator, or activated Frizzled 5 (FZD5) and LDL receptor-related protein 5 (LRP5), positive regulatory factors of β-catenin signaling, to up-regulate the level of β-catenin. The direct regulatory effect of KLF4 on the β-catenin pathway in GDD needs to be further studied.

In conclusion, we verified the critical role of miR-34c-5p in abnormal bone formation of GDD in vitro and in vivo. Additionally, this study identified *Klf4* as a novel target gene of miR-34c-5p. Overexpression of miR-34c-5p effectively attenuated enhanced osteogenesis through directly inhibiting KLF4 and β-catenin signaling. Notably, the application of AAV9-miR-34c-5p rescued abnormal bone phenotype of GDD, including decreasing serum P1NP level, reducing cortical bone thickness, and enhancing biomechanical properties. Our current study revealed that aberrant miR-34c-5p/KLF4/β-catenin signaling is the crucial mechanism of GDD occurrence and further provides new insights into the clinical treatment of GDD by targeting miR-34c-5p.

## 4. Materials and Methods

### 4.1. Subsection Animal

*Ano5*^−/−^ mice were bred from a C57BL/6N background, constructed using CRISPR/Cas9 technology, and a total of 833 bases at both ends of exon 11 and 12 of the *Ano5* gene were clipped. Skeletal characteristics and osteoblastic abilities of the GDD mice have been published [7]. All the mice were housed in a specific pathogen-free (SPF) facility in Beijing Stomatological Hospital Affiliated with Capital Medical University at room temperature (24 ± 2) °C, with 12 h daylight/dark cycle and access to laboratory standard food and water. Animal feeding and experimental procedures were approved by the Animal Care and Ethics Committee of Beijing Stomatological Hospital, Capital Medical University (KQYY-202012-005).

### 4.2. Cell Culture

Two genotypes (*Ano5^+/+^, Ano5^−/−^*) of mouse calvarial osteoblasts (mCOBs) were isolated from the skulls of newborn mice in the same litter. The skulls were placed in a digestive solution with a 1:1 mixture of 0.05% pancreatic enzyme and 0.15% type I collagenase (Sigma Aldrich, St. Louis, MO, USA) and then digested in an incubator at 37 °C for 4 cycles. Each cycle lasted 15 min, and the cells of the second to fourth cycle were collected for primary cell culture. The culture medium was DMEM containing 20% fetal bovine serum (FBS) and 1% streptomycin/penicillin mixture (Gibco-Invitrogen, Grand Island, NY, USA). The cells were placed in large dishes at 37 °C in a humidified atmosphere of 5% CO_2_. After cells reached confluence, they were passaged. Then the medium was changed to α-MEM (Gibco-Invitrogen) containing 10% FBS and 1% streptomycin/penicillin mixture (Gibco-Invitrogen). MC3T3-E1 Subclone was bought from OriCell (Cyagen Biosciences Inc., Guangzhou, Guangdong, China). Cultivation medium was changed every 2 days.

For osteoblast differentiation, mCOBs and MC3T3-E1 were cultured in α-MEM containing 10% FBS, 1% streptomycin/penicillin mixture, 4 μg/mL dexamethasone, 50 mg/mL ascorbic acid (Sigma Aldrich), and 4 mM β-glycerophosphate (Sigma Aldrich). IWR-1 (Selleck Chemicals, Houston, TX, USA) was used to inhibit Wnt/β-catenin signaling in *Ano5*^−/−^ mCOB.

### 4.3. Quantitative Reverse Transcription-Polymerase Chain Reaction (qRT-PCR)

Total RNA was extracted using Trizol (CWBio, Beijing, China), and the concentration and purity of RNA were detected using Nanodrop2000. Reverse transcription of RNA into cDNA was completed by the PrimeScript™ RT reagent Kit (Takara, Kusatsu, Shiga, Japan) according to the manufacturer’s instructions. Gene expression levels were detected using the Ultra SYBR Mixture fluorescent quantitative PCR kit (CWBio) with 1 µL cDNA as a template, amplified by a real-time quantitative PCR system (Bio-Rad, Hercules, CA, USA). The PCR process involves initial enzyme activation at 95 °C for 10 min, followed by 40 cycles of denaturation at 95 °C for 30 s, annealing at 60 °C for 60 s, and extension at 72 °C for 30 s. Gene expression data were normalized to the internal reference of β-actin (for mRNA) or U6 (for miRNA) expression using the delta-delta comparative threshold cycle (2^−ΔΔCt^) method. The primers were listed in Supplemental Tables S1 and S2, and the primers of miR-34c-5p and U6 were supplied by RiboBio (RiboBio, Guangzhou, Guangdong, China).

### 4.4. Western Blot Analysis

The protein concentration was measured using QC Colloidal Coomassie Stain (Bio-Rad). Proteins were separated using SDS-Pre-cast PAGE gels (NCM Biotech, Suzhou, Jiangsu, China) and transferred onto polyvinylidene difluoride (PVDF) membranes (Merck Millipore, Darmstadt, Germany). PVDF membranes were blocked with 5% skim milk in TBST (Bio-Rad) for one hour and incubated with primary antibodies on a shaker overnight at 4 °C. PVDF membranes were incubated with horseradish peroxidase (HRP)-coupled secondary antibodies at a dilution of 1:5000 for one hour at room temperature and visualized using the ChemiDoc imaging system (Bio-Rad). The band intensity of target proteins was normalized to the intensity of the bands of β-actin. All primary antibodies used were listed in Supplementary Table S3.

### 4.5. Transfections

The mimics or inhibitor of miR-34c-5p or their negative control (GeneChem, Shanghai, China) was transfected into MC3T3-E1 at MOI = 80 and mCOBs at MOI = 50 when the cells grew to 30% confluence. The transfection medium was refreshed 16 h after transfection. Puromycin (GeneChem) at 2.5 ug/mL was added to the complete medium for screening, and after 4 days, when an intracellular fluorescence signal was detected, the stably transformed cells were obtained.

The siKLF4 or negative control (Genomeditech, Shanghai, China) was transfected into mCOBs at 100 nM using GMTrans Liposomal Transfection Reagent (Genomeditech). The transfection medium was refreshed 6 h after transfection. The siRNA sequences were listed in Supplemental Table S4.

### 4.6. Cell Proliferation Assay

The proliferation ability of mCOBs was measured using the Cell Counting Kit-8 (CCK-8) assay (Beyotime, Shanghai, China) according to the manufacturer’s instructions. After transfection, cells were seeded into 96-well plates at a density of 2 × 10^3^ cells/well and incubated at 37 °C for 6 h to facilitate their adherence. The time at this point was set as hour 0. At the indicated time points (0, 24 h, 48 h, and 72 h), the medium was completely replaced with serum-free medium, and 10 μL CCK-8 solution was added to each well. Then the cells were incubated at 37 °C for 2 h, and the OD value was measured at 450 nm on a microplate reader.

### 4.7. Alkaline Phosphatase (ALP) Staining and Activity Assay

Cells were seeded into 12-well plates at a density of 1 × 10^5^ cells/well and underwent osteogenesis induction for 5 days. After fixation with paraformaldehyde at 4% concentration, the ALP staining was performed with BCIP/NBT substrate solution (Beyotime). Then ALP activity was examined using an Alkaline Phosphatase Assay Kit (Nanjing Jiancheng, Nanjing, Jiangsu, China) according to the manufacturer’s protocol. The absorbance was examined at 405 nm.

### 4.8. Alizarin Red Staining

Cells were seeded into 12-well plates at a density of 1 × 10^5^ cells/well and underwent osteogenesis induction for 21 days. After fixation with paraformaldehyde at 4% concentration, the Alizarin red staining was performed with Alizarin Red S (Beyotime) for 20 min, then washed with water.

### 4.9. Dual-Luciferase Reporter Assay

The potential target genes for miR-34c-5p were predicted using an online database (http://www.targetscan.org/ accessed on 20 May 2025). Wild-type (WT) and mutant (MUT) 3′UTR luciferase reporter gene vectors for *Klf4* were constructed by Genechem. The miR-34c-5p mimics or mimics control were co-transfected with the constructed WT or MUT luciferase reporter gene vectors into HEK-293T cells using the GMTrans Liposomal Transfection Reagent (Genomeditech). Detection was performed 48 h after cell transfection using the Dual-Luciferase Reporter Gene Detection Kit (Yeasen, Shanghai, China).

### 4.10. Immunofluorescence

Cells were seeded in 12-well plates and immunofluorescence staining was performed at 0 and 7 days of osteogenic induction. After discarding the medium, the cells were fixed with 4% paraformaldehyde (Servicebio, Wuhan, Hubei, China) for 20 min, then blocked with quick immunofluorescence blocking solution (Beyotime) for 15 min, and incubated with primary antibody overnight at 4 °C. On the second day, the cells were incubated with Cy3-labeled secondary antibody (Abclonal, Wuhan, Hubei, China) for 1.5 h at room temperature in the dark shaker. After washing with PBS, the cells were incubated with phalloidin (Abcam, Cambridge, UK) for 45 min at 37 °C and dapi (Sigma) for 10 min at room temperature. The fluorescence was observed and photographed under a fluorescence microscope (Zeiss, Oberkochen, Baden-Württemberg, Germany). We selected 6 fields of view in each group of cells, and the ROI was all cells under that field of view. The average fluorescence intensity of the target protein was calculated and each experiment was repeated 3 times. The ratio of the fluorescence intensity of the target protein to the fluorescence intensity of F-actin and the number of nuclei in the same area was calculated as the average fluorescence intensity, and statistical analysis was performed.

### 4.11. In Vivo AAV-miR-34c-5p-Treatment

We performed painless AAV injection in mice under anesthesia. Isoflurane (3% for induction and 1.5% for maintenance) was administered via a precision vaporizer to ensure adequate anesthesia throughout the procedure. Animals showing signs of severe pain, distress, or morbidity were euthanized promptly.

Healthy, age-appropriate *Ano5^+/−^* female and male mice were mated at a ratio of 2:1 to obtain littermates. When the mice reached 8 weeks of age, healthy male *Ano5*^+/+^ and *Ano5*^−/−^ mice with similar body weights were selected for the experiment. We obtained 7 *Ano5*^+/+^ mice and randomly selected 17 *Ano5*^−/−^ mice that met the criteria. The mice were divided into three groups: the *Ano5*^−/−^ group (*n* = 6), the *Ano5*^−/−^ + AAV9-miR-34c-5p group (*n* = 5), and the *Ano5*^−/−^ + NC group (*n* = 6). Then the mice were randomly assigned to cages.

AAV9-mmu-mir-34c-5p was synthesized at a titer of 2.49E + 13 v.g./mL, and the virus was diluted to 5E + 12 v.g./mL with PBS according to the manufacturer’s instructions (GeneChem). The 8-week-old male mice of *Ano5*^+/+^ and *Ano5*^−/−^ genotypes were randomly selected to be grouped. We injected the AAV virus or control into the knee joints and the lingual side of mandible of *Ano5*^−/−^ mice, and the amount of virus injected into each site was 5 ul. After 8 weeks of observation, the mice were euthanized.

Animals were euthanized by CO_2_ asphyxiation at a flow rate of 20% chamber volume per minute, followed by secondary confirmation of death via thoracotomy. All steps were performed using a blinded approach, with each step carried out by different experimenters.

### 4.12. Enzyme-Linked Immunosorbent Assay (ELISA)

Blood was obtained from retro-orbital blood sampling of 16-week-old male mice. After one hour at room temperature, and 5 min centrifugation at 3000 rpm, the serum was isolated. ELISA kits were used to measure the levels of serum alkaline phosphatase (ALP) (Nanjing Jiancheng) and procollagen type I N-terminal peptide (P1NP) (Elabscience, Wuhan, Hubei, China), according to the manufactures’ instructions.

### 4.13. Skeletal Analysis

The bones of 16-week-old mice were harvested, fixed in 4% paraformaldehyde, and changed to 70% alcohol after 24 h. The femurs, tibias, and mandibles were evaluated using micro-computed tomography (μCT) (Inve on CT, Siemens, Germany). Dataviewer (Bruker, Billerica, MA, USA), CTAn (Bruker), and Mimics Medical 21.0. (Materialise, Leuven, Belgium) were used to analyze the histomorphological characteristics of bone. We selected 100 layers of images (from 50 layers below the growth plate) as the region of interest for trabecular and 100 layers of images (from 450 layers below the growth plate) for the cortex. Images of tibias and femurs were captured, and data were analyzed in both the coronal plane and cross sections. We calculated the bone mineral density (BMD) and the bone mineral content (BMC) according to the Hounsfield unit (HU) value using calibrated CT data and an interactive medical image control system. The trabecular bone BMD, BMC, volume/total volume BV/TV (%), trabecular number, and trabecular spacing (μm) were also measured. And we calculated the thickness of the bone cortex at the 450 layers below the growth plate. When observing the mandible in the coronal plane, the central section of the first molar is chosen as the reference plane. Subsequently, 20 layers of images surrounding the reference plane are selected as the region of interest (ROI) for the purpose of measuring the cortical bone and trabecular bone and carrying out statistical analysis.

### 4.14. Frozen Section Preparation of Bone Tissue

Fresh bone samples were fixed in 4% PFA (Servicebio, Wuhan, Hubei, China) for 24 h, decalcified in 10% EDTA (pH 7.4) (Servicebio) for 7–14 d, cryoprotected in 30% sucrose, embedded in OCT, and frozen in liquid nitrogen. Sections (8–10 μm) were cut using a cryostat at −20 °C, mounted on poly-L-lysine-coated slides (Citotest, Nanjing, Jiangsu, China), air-dried, and stored at −80 °C for further analysis.

### 4.15. H&E Staining of Bone Tissue

Decalcified bone samples were embedded in paraffin and sectioned at 5 μm thickness. Sections were deparaffinized, rehydrated, and stained with hematoxylin for 5–10 min, followed by differentiation in 1% acid alcohol and bluing in 0.1% ammonia water. Counterstaining was performed with eosin for 1–2 min. After dehydration and clearing, slides were mounted with neutral balsam (Zhongshan Golden Bridge Biotechnology, Beijing, China) and imaged under a light microscope (Olympus Corporation, Tokyo, Japan).

### 4.16. Three-Point Bending Test

Tibias were collected from 16-week-old male mice in saline solution and stored at −80 °C. The three-point bending test was performed using a universal material testing machine (AG-X Plus, Shimadzu, Japan) at the midshaft of tibias with a displacement rate of 1 mm/min until the bone fractured with the force and displacement acquired digitally. The tibia was fixed to the lower bracket at a fixed distance of 1 cm. Structural properties, including maximum load and fracture load, were calculated. The maximum stress and failure stress of each tibia were calculated according to the corresponding load and tibial diameter.

### 4.17. Statistical Analysis

All results were confirmed in at least three independent experiments. Data analysis was conducted using GraphPad Prism 9.0 (GraphPad Software, San Diego, CA, USA). The data are presented as the mean ± standard deviation (SD) derived from at least three independent experiments. Statistical differences between two groups were determined using Student’s *t*-test. For comparisons involving three or more groups, one-way ANOVA was performed, followed by Tukey’s post hoc test for multiple comparisons. *p*-Values < 0.05 were considered statistically significant.

## Data Availability

The data that support the findings of this study are available from the corresponding author upon reasonable request.

## Supplementary Materials

The following supporting information can be downloaded at: https://www.mdpi.com/article/10.3390/ijms26115267/s1.

## Abbreviations

The following abbreviations are used in this manuscript:

GDD	Gnathodiaphyseal dysplasia
*Ano5*	*Anoctamin 5*
ALP	alkaline phosphatase
AAV	adeno-associated virus
P1NP	N-terminal peptide of type I collagen
mCOBs	calvaria-derived osteoblasts
OCN	osteocalcin
RUNX2	runt-related transcription factor 2
KLF4	Krüppel-Like Factor 4
BMC	bone mineral content
BMD	bone mineral density
