# Supplementary material for: Ano5 Deficiency Leads to Abnormal Bone Formation via miR-34c-5p/KLF4/β-Catenin in Gnathodiaphyseal Dysplasia
Source: Int J Mol Sci. 2025 May 30;26(11):5267. doi: 10.3390/ijms26115267 (PMC12155156; doi:10.3390/ijms26115267)
Supplement: Supplementary file 1 [file ijms-26-05267-s001.zip › ijms-3556758-supplementary.pdf]

Supplementary Material

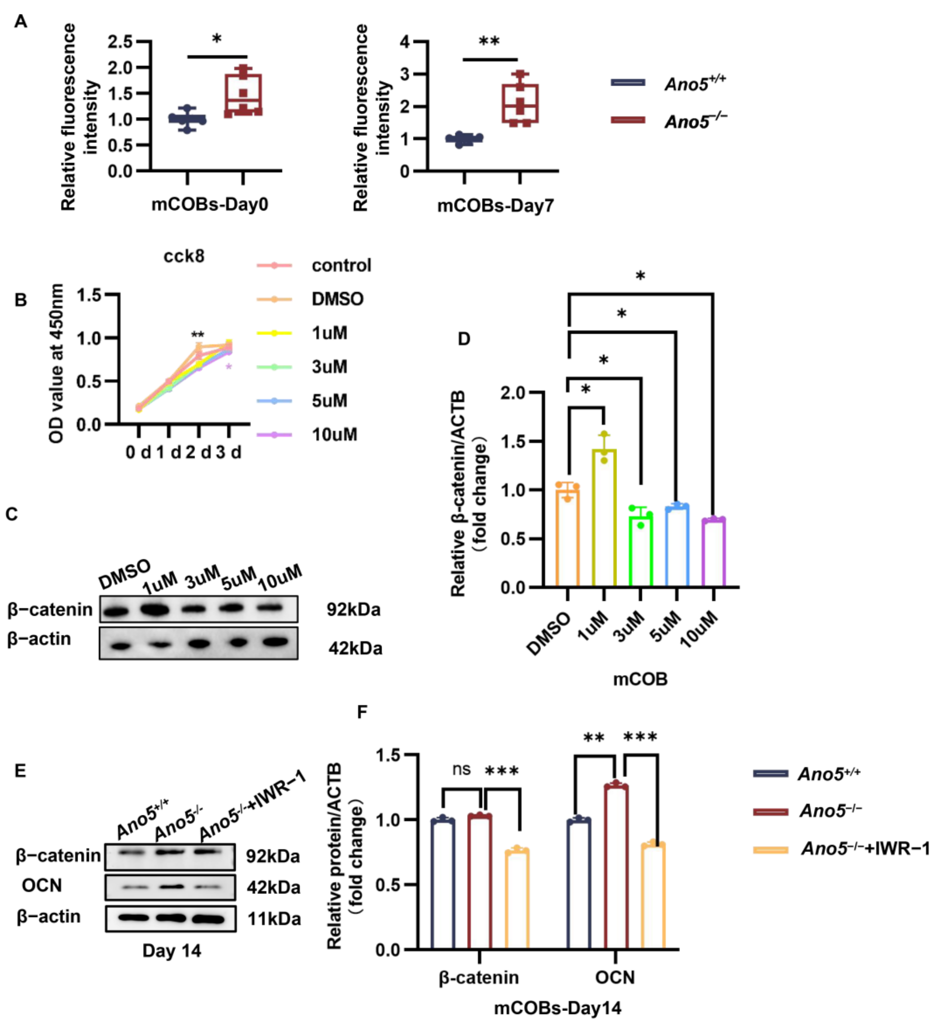

Figure S1. miR-34c-5p/KLF4 regulated the β-catenin pathway and influenced osteogenic differentiation. (A) The average fluorescence intensity of total β-catenin in mCOBs was analyzed. (B-D) The most suitable concentration of IWR-1 was selected using CCK-8 and Western blot. (E, F) IWR-1 decreased the expression of OCN in *Ano5*$^{-/-}$ mCOBs at 14 days of osteogenic induction. *P<0.05, **P<0.01, ***P < 0.001, ns: no significant.

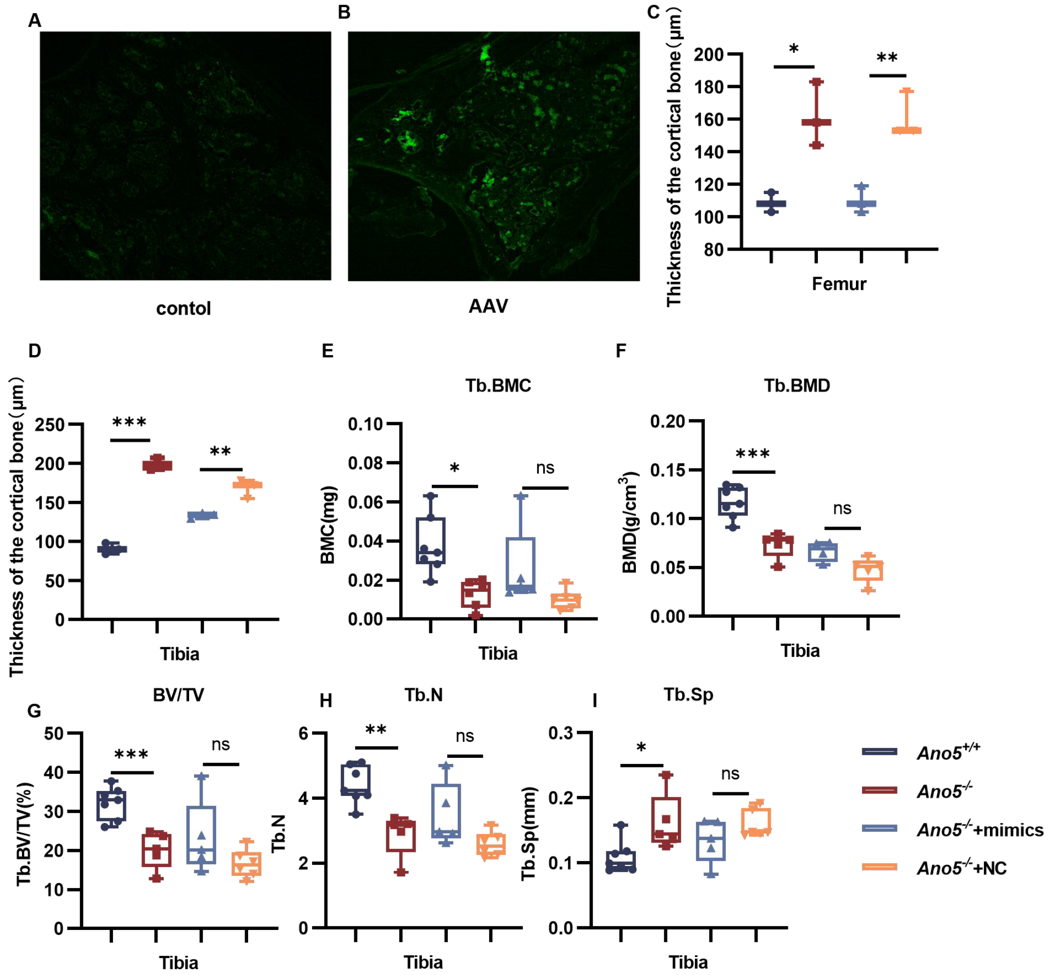

Figure S2. AAV9-miR-34c-5p treatment didn't rescued the tibia trabecula. (A, B) The femurs of mice in the AAV transfection group had stronger fluorescence expression than those in the control group. (C) The cortical bone of the femur was analyzed in H&E staining. (D) The cortical bone of the tibia was analyzed in H&E staining. The tibia-relative parameters were analyzed, including the BMC (E), BMD (F), (G), number (H), and separation of the trabecula (I). Data were presented as box plots with an indication of the median; whiskers represented min to max values and showed all points. Each dot represented a single animal. n=7 for *Ano5*$^{+/+}$mice, n=6 for *Ano5*$^{-/-}$mice, n=5 for *Ano5*$^{-/-}$+miR-34c-5p mice, n=6 for *Ano5*$^{-/-}$+negative control mice. **P*<0.05, ***P*<0.01, ****P*<0.001, and ns: no significant.

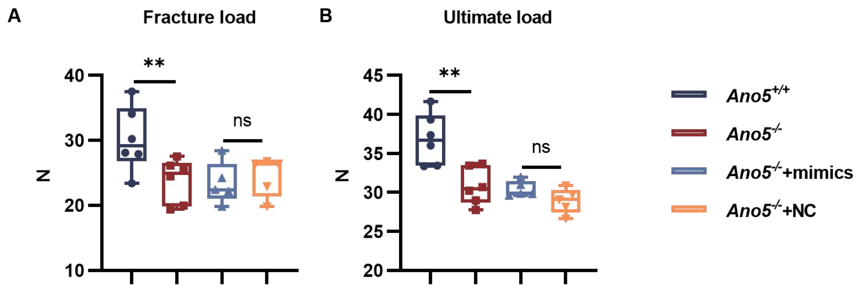

Figure S3. AAV9-miR-34c-5p treatment didn't change the fracture force and ultimate force of the tibia. Three-point bending test showed the fracture force (A) and ultimate force of the tibia (B). Data were presented as box plots with an indication of the median; whiskers represented min to max values and showed all points. Each dot represented a single animal. *n*=7 for *Ano5*$^{+/+}$mice, *n*=6 for *Ano5*$^{-/-}$mice, *n*=5 for *Ano5*$^{-/-}$+miR-34c-5p mice, *n*=6 for *Ano5*$^{-/-}$+negative control mice. $^{**}P<0.01$， ns: no significant.

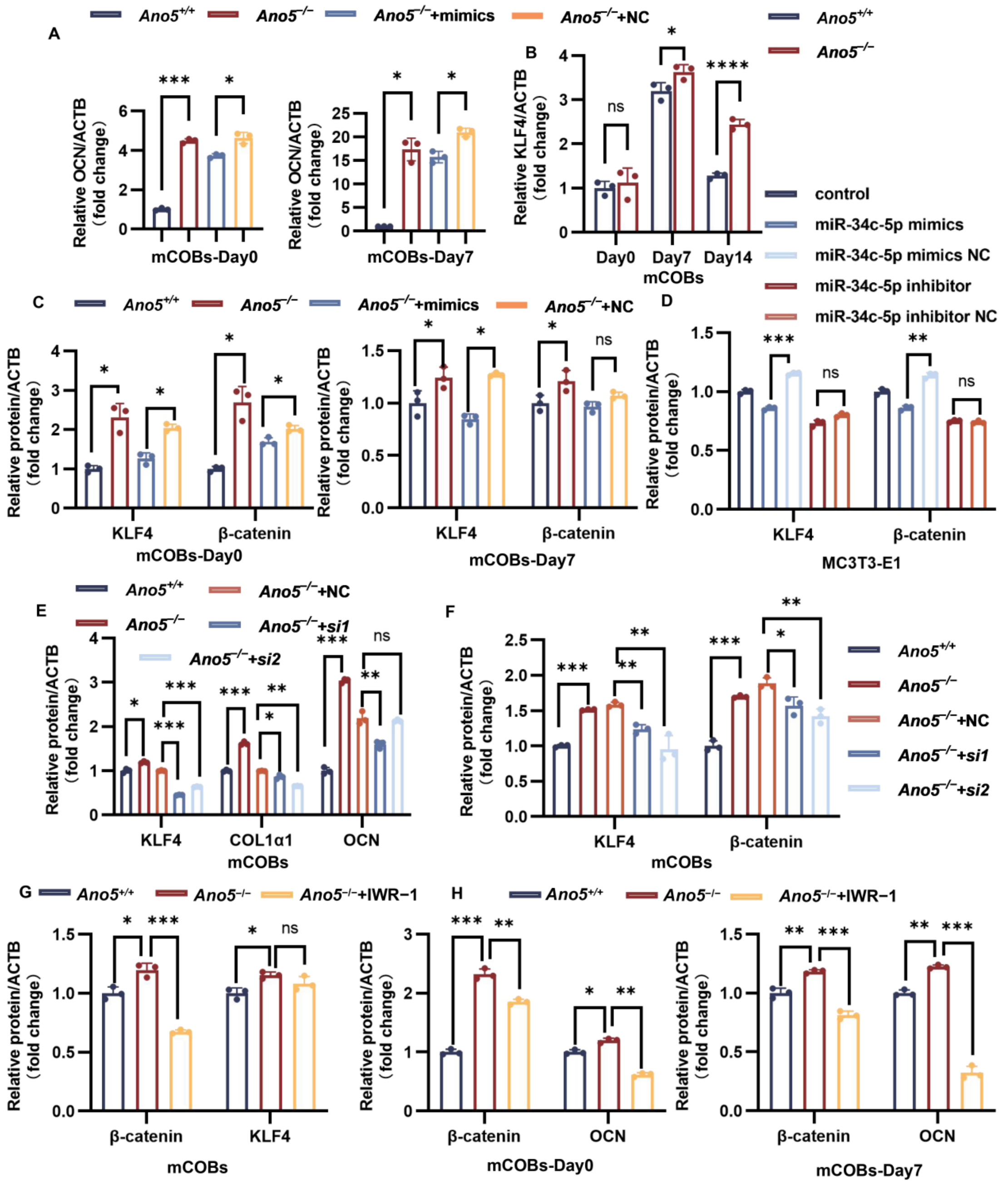

Figure S4. Quantitative analysis of the gray value of Western blot bands. A. Quantitative analysis of the expression level of OCN protein in Figure 2F. B. Quantitative analysis of the expression level of KLF4 protein in Figure 3C. C. Quantitative analysis of the expression level of KLF4 and β-catenin protein in Figure 2H. D. Quantitative analysis of the expression level of KLF4 and β-catenin protein in Figure 2J. E. Quantitative analysis of the expression level of KLF4, COL1α1 and OCN protein in Figure 4F. F. Quantitative analysis of the expression level of KLF4 and β-catenin protein in Figure 5D. G. Quantitative analysis of the expression level of KLF4 and β-catenin protein in Figure 5E. H. Quantitative analysis of the expression level of β-catenin and OCN protein in Figure 5F. **P*<0.05, ***P*<0.01, ****P*<0.001, **** ***P*<0.0001,**ns: no significant.

Supplementary Material

Supplementary Table S1. Primers used for PCR

Genes	Forward primer sequences:	Reverse primer sequences
β-actin	GTGACGTTGACATCCGTAAAGA	GCCGGACTCATCGTACTCC
Ocn	GAACAGACAAGTCCCACACAGC	TCAGCAGAGTGAGCAGAAAGAT
Bsp	AATGGAGACGGCGATAGTTCCG	GGAAAGTGTGGAGTTCTCTGCC
Runx2	TCCTGTAGATCCGAGCACCA	CTGCTGCTGTTGTTGCTGTT
Klf4	GTGCCCCGACTAACCGTTG	GTCGTTGAACTCCTCGGTCT
Axin2	TGACTCTCCTTCCAGATCCCA	TGCCCACACTAGGCTGACA
c-Myc	ATGCCCCTCAACGTGAACTTC	CGCAACATAGGATGGAGAGCA

Supplementary Table S2. miRNA primers used for PCR

Primer	Catalog number
**Bulge-LoopTM mmu-miR-34c-5p RT Primer**	ssD9831040219
**Bulge-LoopTM mmu-miR-34c-5p Forward Primer**	ssD9831042219
**Bulge-LoopTM miR-Reverse Primer**	ssD089261711
**Bulge-LoopTM U6-RT Primer**	ssD0904071008
**Bulge-LoopTM U6-Forward Primer**	ssD0904071006
**Bulge-LoopTM U6-Reverse Primer**	ssD0904071007

Supplementary Table S3. Antibodies Used for Western blot and IF

Antibody	Catalog number	Brand
**Anti-OCN mAb**	DF12303	Affinity Biosciences
**Anti-KLF4 mAb**	214666	Abcam
**Anti-β-catenin mAb**	51067-2-AP	proteintech
**Anti-β-actin Rabbit mAb**	Ac026	ABclonal
**Anti-COL1A1 Rabbit pAb**	A1352	ABclonal
**Cy3-conjugated Goat anti-Rabbit IgG (H+)**	AS007	ABclonal

Supplementary Table S4. siRNA sequences used for gene silencing

Gene	Sense sequences	Antisense sequences
**KLF4-1**	CGGUCAUCAGUGUUAGCAA	UUGCUAACACUGAUGACCG
**KLF4-2**	CACCCACACUUGUGACUAU	AUAGUCACAAGUGUGGGUG
